# Visuomotor predictors of interception

**DOI:** 10.1371/journal.pone.0308642

**Published:** 2024-09-16

**Authors:** Inmaculada Márquez, Mario Treviño

**Affiliations:** 1 Departamento de Ciencias Médicas y de la Vida, Centro Universitario de la Ciénega, Universidad de Guadalajara, Ocotlán, México; 2 Laboratorio de Conducta Animal, Departamento de Psicología, Centro Universitario de la Ciénega, Universidad de Guadalajara, Ocotlán, México; 3 Laboratorio de Plasticidad Cortical y Aprendizaje Perceptual, Instituto de Neurociencias, Universidad de Guadalajara, Guadalajara, Jalisco, México; The Ohio State University, UNITED STATES OF AMERICA

## Abstract

Intercepting moving targets is a fundamental skill in human behavior, influencing various domains such as sports, gaming, and other activities. In these contexts, precise visual processing and motor control are crucial for adapting and navigating effectively. Nevertheless, there are still some gaps in our understanding of how these elements interact while intercepting a moving target. This study explored the dynamic interplay among eye movements, pupil size, and interceptive hand movements, with visual and motion uncertainty factors. We developed a simple visuomotor task in which participants used a joystick to interact with a computer-controlled dot that moved along two-dimensional trajectories. This virtual system provided the flexibility to manipulate the target’s speed and directional uncertainty during chase trials. We then conducted a geometric analysis based on optimal angles for each behavior, enabling us to distinguish between simple tracking and predictive trajectories that anticipate future positions of the moving target. Our results revealed the adoption of a strong interception strategy as participants approached the target. Notably, the onset and amount of optimal interception strategy depended on task parameters, such as the target’s speed and frequency of directional changes. Furthermore, eye-tracking data showed that participants continually adjusted their gaze speed and position, continuously adapting to the target’s movements. Finally, in successful trials, pupillary responses predicted the amount of optimal interception strategy while exhibiting an inverse relationship in trials without collisions. These findings reveal key interactions among visuomotor parameters that are crucial for solving complex interception tasks.

## Introduction

Intercepting moving targets is a crucial ability in human behavior as it impacts various aspects of our daily lives, such as sports, gaming, and other common activities [1, 2]. Tasks like catching a ball, driving a car, and navigating through crowded places rely on successfully intercepting (and avoiding) moving objects. The complexity of visuomotor coordination when solving these tasks influences our ability to interact and adapt to dynamic environments [3–5]. Visuomotor coordination involves neuronal activity in visual and motor-related brain areas. Some authors suggest that such coordination triggers a sequence of events, organized in parallel and processed hierarchically [6]. This specialized coordination enables individuals to continuously adapt their motor commands based on feedback, thus refining accuracy over time. Therefore, the efficiency of target interception is influenced by a combination of strategy, kinematic ability, and environmental conditions. Some theories suggest that interception strategies are built upon cues such as the target’s position and its kinematic derivatives [7]. An intriguing possibility emerges from the potential connection between distinct cognitive and adaptive strategies that may underlie interceptive behavior.

Various operational control models can be employed for interception. In pursuit guidance, for example, the velocity vector of the approach consistently aligns with the target. This often results in a tail-chase towards the end of the target’s trajectory, which can require longer chasing trajectories than other guidance laws. Indeed, predictive elements can be incorporated into these guidance laws to enhance their effectiveness [8]. Predictive models are based on the idea of predicting the future position of the target. This model is more complex than simple pursuit but potentially more efficient, as it can anticipate the target’s movements and intercept it using a shorter path to its destination. Numerous predictive models exist, sharing a common foundation: evaluating the target’s current position and velocity to predict its future location. Different interception modes, which use pre-existing information and real-time adjustments in varying ways to determine the interception point and trajectory, have been discussed elsewhere [9–14].

Visual processing plays a crucial role in interception by contributing to the estimation of the target’s position, velocity, and timing prediction. Additionally, the sudden occlusion of the target just before collision increases interception variability, highlighting the contribution of visual inputs for making predictions in these tasks [15]. Eye movements provide essential cues for interception and can involve anticipatory saccades reflecting the prediction of future target locations [16–18]. They provide visual feedback for hand movements and contribute to motion prediction, a process that can be modulated by altering the variability of the target’s trajectory [19]. In interception tasks, individuals adjust their hand movements based on the target’s position, responding to positional changes within approximately 100 ms [20]. Similarly, saccades, particularly those occurring around 100 ms before interception, are crucial for timing and accuracy in such dynamic tasks. Active gaze movements, as opposed to fixating on a point, reduce errors, making them a beneficial strategy for enhancing visuomotor coordination [21, 22]. Pupillary responses, often linked to changes in luminance levels, are also associated with decision-making processes [23–25]. For instance, research indicates that cognition can regulate pupil size to selectively filter visual information for specific objectives, influencing visual perception. Additionally, pupil dilation has been proposed as a psychophysiological measure for dynamic difficulty adjustment. Pupil responses correlate with game difficulty, suggesting that pupil dilation can be used to adaptively adjust task difficulty, ultimately enhancing user engagement and performance [26]. How cognition impacts visual perception through changes in pupil size is a topic of ongoing exploration [27].

Despite acknowledging their critical role in guiding motor commands during visuomotor tasks, substantial gaps exist in understanding how ocular movements relate to interception strategies under diverse task conditions and their precise role in guiding such strategies. Moreover, the potential interplay between gaze and interceptive strategies, influenced by visual and motion uncertainty, remains relatively unexplored. For example, it is unclear how task demands, individual preferences, and eye movement strategies influence the specific relationship between pupillary responses and moving target interception. In this study, we investigated the visuomotor strategies underlying interceptive actions in a virtual task with participants using a joystick to chase a moving target along unpredictable trajectories. Our objective was to assess how alterations in target speed (and directional uncertainty) impact visuomotor responses and target interception. We hypothesized that dynamic interactions among eye movements, pupil size, and interceptive hand movements should be critical for effectively intercepting a moving target under varying conditions of visual and motion uncertainty. Alternatively, without such adjustments, we would expect participants to fail in adapting their visuomotor strategies to changes in target speed and directional uncertainty, resulting in unsuccessful interception attempts. To quantify the impact of task parameters on interceptive performance, we employed a geometric model to distinguish between manual pursuit and interception trajectories. Using eye-tracking data, we also characterized the gaze adjustments based on the target’s position and velocity. Finally, we investigated pupillary responses and their correlation with ‘collision’ trials, examining their dynamics as the user-controlled dot approached and touched the computer-controlled dot. Our findings carry potential implications for interventions in visuomotor coordination impairments, advancements in robotics, and human-computer interfaces. They also contribute to our understanding of how humans engage with the world at the millisecond scale.

## Materials and methods

### Participants

We conducted experiments with 248 adults (139 women, 109 men) aged 18.9 to 26.2 (mode = 23). The testing period spanned from February 10th to August 6th, 2023. An informed, written consent was obtained from all participants. We set specific inclusion criteria for the study: being born at term, and having no reported prenatal, perinatal, or postnatal complications or trauma that could potentially impact nervous system development. By applying these inclusion criteria, we aimed to create a study group of participants whose neurological development could represent a ‘typical’, neurologically healthy population. None of the participants had a diagnosed psychiatric, neurological, or neurodevelopmental disorder nor a history of substance use. All participants included in the analysis were right-handed (*i*.*e*., using their right hand for writing; 5 left-handed participants were excluded from the analysis) and had either normal vision or vision corrected to normal, as assessed by the Snellen test. Participants received written instructions for completing questionnaires, including demographic information, and for performing the task. Participation was voluntary and did not involve any monetary incentives. Participants were selected using a non-probability convenience sampling method due to their accessibility and proximity to the researchers. The sample size of our experiments (see below) allowed a robust analysis of individual differences, consistent with previous studies conducted in our laboratory [28]. All procedures were non-invasive, complied with our country’s local guidelines and regulations, and were approved by the ethics committee of the Instituto de Neurociencias, Universidad de Guadalajara, México: ET102021-330 and ET122023-382.

### Visuomotor task

We described the task in detail previously [29]. Participants sat 60 cm from a 27-inch computer monitor (1920 x 1080 pixels, 60 Hz). They were engaged in a visuomotor task involving chasing a computer-controlled black dot by means of a user-controlled white dot (0.3° of visual angle each). Participants utilized a joystick, held in their dominant hand, to control the white dot with a third-person viewpoint. The task involved chasing and colliding with the computer-controlled black dot, referred to as the ’target,’ which followed piecewise linear trajectories (S1A, S1B Fig in S1 File). We presented both dots in a circular arena that covered the entire height of the monitor, set against a 50% gray background. The circular geometry of our task requires a control mechanism capable of moving in all directions with uniform effort. This factor led us to choose a joystick for tracking participants’ responses, as it ensures consistent effort exertion irrespective of the chosen direction. We calibrated the joystick daily and recorded its relative position, allowing participants to vary and control the speed of the white dot. The angular gradient of the joystick’s tilt determined the speed, which was linearly proportional to the deviation from the vertical axis. At the start of each trial, we positioned the participant’s dot at the center of the screen, and the target appeared at a random location along the circular limits of the arena. A ‘collision’ involved a successful trial where both dots collided within 5 seconds, the designated maximum trial duration. We chose the term collision based on its physics origin, where it implies the convergence of participant and computer dots. The term collision accurately reflects dot convergence, regardless of intended interception outcomes, recognizing that not every collision leads to successful interception and allowing for random or chance occurrences. Trials that did not result in a collision were categorized as unsuccessful chasing trials. We provided auditory feedback (pure tones) to indicate successful or unsuccessful trials. Following each trial, we required the participants to return the joystick to its initial centered position before initiating the subsequent trial.

We manipulated task difficulty by controlling three main parameters. First, we varied the target’s direction using random numbers within an angular range (AR) of ±75° (uniform distribution). Second, we implemented three ‘fixed-direction intervals’ (FDI) of 100 ms, 200 ms, or 500 ms, determining how long the target’s movement direction remained constant before changing to a new random angle within the specified range. Hence, the AR influenced the predictability of the target’s direction changes, whereas the FDI controlled the frequency of these changes (S1A Fig in S1 File). Lastly, we used multiple target speeds (*v*_*T*_): 10°/s, 20°/s, 30°/s, 40°/s, 50°/s, and 60°/s. The joystick did not influence the velocity or movement of the target. Instead, the target velocity was controlled by the experimenter and remained constant during the entire trial, although it could vary between trials. If the target reached the borders of the circular arena, we produced a bounce using the law of reflection: taking the angle of incidence plus the corresponding angular variability specified for each trial (±75°). In a set of experiments, we examined how the dynamics of chasing trials were influenced when either the user’s or target’s dot temporarily vanished from view. Thus, in these occlusion experiments, some trials involved masking one of the two dots by switching their contrast to 0% (*i*.*e*., making it invisible) at different randomly permuted inter-dot distances (IDD). With this procedure, we investigated how participants engaged with the moving target, and whether they relied on predictive signals or anticipated states, even when confronted with the lack of visual information. We instructed the participants to maintain stillness and minimize head motion while allowing them to move their gaze during the experiment.

To ensure engagement and optimal task performance, we used a maximum of 900 trials per session, with one or two resting periods (each lasting 5 minutes) [28, 30]. Experimental sessions had a duration of less than an hour. We made six experiments (E_1_-E_6_) due to the constraints of each session’s duration. Furthermore, some measurements utilized an eye tracker while others did not, requiring additional experiments for the analyses presented in our Results section. E_1_ and E_2_ explored the impact of different *v*_*T*_ values on the interception task. E_1_ tested trials with fixed directional intervals (FDI) of 100 or 200 ms, while E_2_ tested trials with an FDI = 500 ms. E_3_ and E_6_ involved visual occlusion with *v*_*T*_ of 30°/s and 10°/s, respectively. In these experiments, either the user or the target were occluded at four different IDD values using permutations of occlusion conditions. E_4_ explored various permuted FDI values (FDI: 100 ms, 300 ms, 400 ms, 500 ms, 1000 ms, 1500 ms) with a *v*_*T*_ = 10°/s, while E_5_ replicated this setup with a *v*_*T*_ = 30°/s. Table 1 details the number of participants in each experiment, a general description of the conditions and repetitions for each condition, and references to the corresponding result illustrations. We programmed the visuomotor task in MATLAB R2022a using the Psychophysics Toolbox extensions MATLAB (MathWorks, Natick, MA, United States; PTB-3 [31, 32].

**Table 1:** Participant distribution and experimental conditions.

Exp.	Experimental condition	Trials per experiment	Repetitions per condition	Total Number of participants	Illustrated in Figure(s):
E_1_	{FDI = 100 ms, FDI = 200 ms}, variable V_T_, AR = ± 75°	540	45 trials/[V_T_*FDI] (15 participants / V_T_)	15/V_T_ (woET)	1
E_2_	FDI = 500 ms, variable V_T_, AR = ± 75°	900	150 trials/V_T_	33 (wET)	1,2,4,6
E_3_	Masking cond. (MC: user or target), FDI = 500 ms, V_T_ = 30°/s, {D0, D3, D5, D10}	800	100 trials/[D*MC]	20 (woET) + 20 (wET)	3,5, S3 Fig in S1 File
E_4_	Variable FDI, V_T_ = 10°/s	900	150 trials/FDI	20 (woET)	S2 Fig in S1 File
E_5_	Variable FDI, V_T_ = 30°/s	900	150 trials/FDI	40 (woET)	S2 Fig in S1 File
E_6_	Masking cond. (MC: user or target), FDI = 500 ms, V_T_ = 10°/s, {D0, D3, D5, D10}	800	100 trials/[D*MC]	20 (woET)	S3 Fig in S1 File

We created six experimental groups (first column: E_1_-E_6_) due to the constraints of each session’s duration (maximum 1 hour). Some experiments utilized an eye tracker (’wET’) while others did not (’woET’), requiring additional experiments for the analyses presented. E_1_ and E_2_ explored the impact of different *v*_*T*_ values on the interception task. E_1_ tested trials with fixed directional intervals (FDI) of 100 and 200 ms, while E_2_ tested trials with an FDI = 500 ms. E_1_ involved a larger number of total participants (90) because it had fewer repetitions per condition compared to E_2_. Additionally, E_1_ included 6 subgroups (15 participants each) where each group’s target speed was fixed. E_3_ and E_6_ involved visual occlusion with *v*_*T*_ of 30°/s and 10°/s, respectively. In these experiments, either the user or the target were occluded at four different IDD values using permutations of occlusion conditions. E_4_ explored various permuted FDI values with a *v*_*T*_ = 10°/s, while E_5_ replicated this setup with a *v*_*T*_ = 30°/s. The last column of the table specifies the corresponding figures where the data is depicted.

### Eye tracking

We utilized eye tracking to investigate the oculomotor correlates of successful interception. We recorded binocular eye movements using a commercial eye tracker (Tobii, Pro Fusion, Tobii Pro SDK for Windows; Stockholm, Sweden) operating at a sampling rate of 60 Hz. We fixed the tracker to the lower part of the monitor. The participants were seated comfortably ensuring we centered the eye tracker headbox with their eye level. We minimized participants’ head movements during eye-tracking experiments using a chin holder with a forehead rest. We used this chin rest to maintain consistent and precise eye-tracking data, minimize artifacts, and enhance overall data quality and experimental control. With two cameras capturing stereo images of both eyes, the tracker measured gaze position, and pupil diameter. Throughout the study, we maintained a consistent luminance level in the room where we conducted our experiments. Before initiating calibration, the operator inspected the eye images to ensure clear visibility of relevant eye features, such as the pupil and corneal reflections (c_0_). The next steps were as follows: first, participants underwent a standard 4-point calibration directly from the eye tracker (Tobii Pro Eye Tracker Manager 2.6.0), with fixation points positioned on the corners of the screen (c_1_). Subsequently, we performed a calibration using the Psychophysics Toolbox, involving four fixation white dots (with an outer diameter of 0.6°) placed on the periphery of the circular arena of our visuomotor task to ensure proper alignment between the eye-tracking data and the visual stimuli projection on the screen (c_2_, S4A Fig in S1 File). A third calibration step involved verifying participants’ smooth pursuit response as they tracked a white dot moving horizontally or vertically along the circular arena at a speed of 10°/s (c_3_, S4B Fig in S1 File).

### Pupillometry

The infrared eye tracker recorded binocular pupil diameters with an average background illuminance of ~100 lux at 60 cm from the monitor [33, 34]. We performed a fourth calibration routine of six cycles of alternating contrast conditions (alternating black and white full screens) for each participant to elicit the pupillary light reflex. We repeated this routine four times, each iteration lasting ~35 seconds, enabling us to characterize the averaged participants’ responses (c_4_, S4C Fig in S1 File). We calculated a binocular average of both pupil diameters and performed a normalization by subtracting the average pupil size and then normalizing the participants’ responses using the range of changes in pupil diameter observed during the calibration routine [35]. We established this range by measuring the spread between the 5th and 95th percentiles of the pupillary light reflex responses. We addressed missing data and blink artifacts (identified using the eye tracker’s software) by employing linear interpolation to fill in the gaps in the dataset. These normalized pupillary diameters represent changes in pupil size during our experimental trials, which we analyzed over time in relation to key events such as the start or end of collision trials. For simplicity, we refer to these traces throughout this manuscript as ’pupillary responses’. We processed all pupillary responses using custom routines written in MATLAB. We divided the experimental sessions that employed eye-tracking into three blocks, with two five-minute breaks in between. Before each block, we performed calibration routines (c_1_-c_4_) to ensure precise tracking throughout the entire session.

### Analysis

We continuously tracked the chasing trajectories, allowing us to extract various parameters from consecutively acquired frames (S1A, S1B Fig in S1 File). These included the location of the dots in Cartesian (and polar) coordinates, the IDD, user velocity (*v*_*U*_), and the absolute angle between the dots. We also calculated the optimal manual pursuit angle (*i*.*e*., simple manual pursuit, involving the ideal direction of manual tracking), which represents the line connecting the participant’s joystick position on the screen to the target, and the optimal interception angle (*ϕ*), representing the shortest path for interception:

$$\phi =sin^{-1}\left(\frac{v_{T}sin\lambda }{v_{U}}\right)$$ (1)

where *v*_*T*_ is the target speed, *v*_*U*_ is the instantaneous user’s speed, and λ is the difference between the optimal manual pursuit angle and the target’s direction [29, 36]. In our context, the term "optimal pursuit angle" specifically refers to the angle formed by the line connecting the user-controlled dot to the target’s position (left panel in S1C Fig in S1 File). Concerning Eq 1, if *v*_*U*_ < *v*_*T*_ sin λ, then this equation has no solution. In such instances, we assigned *ϕ* as NaN (*i*.*e*., ’not a number’). However, if *v*_*U*_ ≥ *v*_*T*_ sin λ, two solutions are possible, and *ϕ* corresponds to the one that results in a decrease in IDD [36] (middle panel in S1C Fig in S1 File). We then calculated the distributions of manual pursuit (*ε*_*pursuit*_) and interception (*ε*_*interception*_) errors by subtracting the observed participants’ directional angles from optimal ones. To quantify the participants’ preference for interception behavior, we calculated the amount of optimal interception strategy, measured as the percentage of the optimal interception angle (%OIA, right panel in S1C Fig in S1 File) using the angular errors obtained from each frame, as follows:

$$\%OIA=\frac{\left|\varepsilon_{pursuit}\right|}{\left|\varepsilon_{pursuit}\right|+\left|\varepsilon_{interception}\right|}$$ (2)

where an %OIA value > 50% indicates that the participants’ directional angles leaned towards interception behavior; conversely, %OIA < 50% suggests a tendency toward manual pursuit behavior [29]. This approach, which evaluates angular errors frame by frame based on immediate and perfect adherence to each strategy, does not consider the intrinsic dynamics of behavior, including the inherent variability and complexity in individuals’ approaches to our task. Furthermore, the %OIA is influenced by IDD, which implies that when IDD is high, the distinction between manual pursuit and tracking becomes less clear as angular gradients decrease, resulting in %OIA → 50%. Conversely, with smaller IDD (closer dots), angular gradients increase, allowing %OIA to effectively differentiate between manual pursuit (%OIA < 50%) and interception (%OIA > 50%) strategies. Random maneuvers in our task result in indiscriminate increases in both *ε*_*pursuit*_ and *ε*_*interception*_. However, as *ε*_*interception*_ does not converge to a low value during these instances, %OIA would tend to be less than 50% for all these frames (relative to *ε*_*pursuit*_). In other words, an %OIA > 50% cannot be attributed to random maneuvers. Therefore, to evaluate the influence of ‘interception-independent’ maneuvers during our task, we generated 100 randomized versions of %OIA (%OIA_rnd_) per trial by assigning random user orientations, derived from a uniform distribution to each frame to establish a statistical baseline. In summary, calculating the %OIA served three main purposes: firstly, to identify deviations from %OIA_rnd_; secondly, to quantify the extent to which participants utilized an ’interception strategy’; and thirdly, to compare the values of this index between collision and non-collision trials. An %OIA greater than 50% indicates that participants’ chasing trajectories approached the optimal interception angles described in Eq 1, particularly as collision time approached.

Our geometric model computed optimal manual pursuit and interception angles for each frame using the positions of both dots, *v*_*U*_, and *v*_*T*,_ and then compared these values with the user’s direction on that frame. Therefore, manual pursuit errors (ε_pursuit_) quantified the angular disparity between the chaser’s current direction and the optimal manual pursuit angle, while interception errors (ε_interception_) measured the angular deviation between the chaser’s direction and the optimal interception angle (S1C Fig in S1 File). We confirmed the sensitivity of these measures to the interception task. In the upper panels of S1D Fig in S1 File, we illustrate examples of ε_pursuit_ and ε_interception_ group frequency distributions captured at two different temporal frames: 750 ms and 50 ms before the actual collision (with *v*_*T*_ = 30°/s, FDI = 100 ms, AR = ± 75°, data from participants from E_1_). The manual pursuit error distributions exhibited directional disparities relative to the optimal interception angle well in advance of the collision, but these differences became smaller just before the collision (not illustrated, Watson-Williams test for equal mean directions; 45 frames ≈ 750 ms before collision: *P* < 0.001; 3 frames ≈ 50 ms before collision: *P* > 0.5, 2816 trials, *n* = 15). To explore the dynamic changes in these error distributions, we gathered the error data at different frame intervals relative to the collision time (or the end of the trial for cases without collision). Additionally, to establish a statistical reference, we generated manual pursuit and interception error distributions based on random directions at the same instances as the empirically observed changes (gray, labeled ‘Random’ in the lower panels from S1C Fig in S1 File). The ε_pursuit_ and ε_interception_ distributions were different from those obtained with random directions, indicating that they could not be explained by chance (Watson-Williams test, pursuit: 45 frames ≈ 750 ms before collision: *P* < 0.001; 3 frames ≈ 50 ms before collision: *P* < 0.0001; interception: 45 frames ≈ 750 ms before collision: *P* < 0.009; 3 frames ≈ 50 ms before collision: *P* < 0.001, *n* = 15). There were similar differences between observed and random directions for all *v*_*T*_ and FDI tested (not illustrated, Watson-Williams test, *P* < 0.05 for all cases).

IDD, %OIA, and pupillary traces are dynamic responses that evolve uniquely depending on participants’ approach towards the target, displaying particular trajectories over time. Therefore, we calculated the area under the curve (AUC, 800 ms before trial end) for IDD, %OIA (using the positive differences between the observed and random OIA traces), and pupillary response traces as proxies to distinguish conditions that reflect participants’ efficiency in intercepting the moving target. We fitted performance data with psychometric curves using the following equation:

$$f\left(x\right)=\frac{L}{1+e^{-\gamma \left[\cdot x-x_{50}\right]}}+o$$ (3)

where *L* is the curve’s maximum value, *γ* is the curve’s logistic growth rate or slope, *x*_*50*_ is the *x* value of the sigmoid’s midpoint, and *o* is the offset of the entire curve [37]. The psychometric fits, depicted in S2C Fig in S1 File, served to identify the critical FDI value at which the %OIA saturation occurred.

Gaze traces were aligned and averaged for each participant and across experiments, with the alignment performed to the beginning or the end of each trial (*i*.*e*., event-triggered averages). No filtering was applied to the eye-tracking data. We incorporated two additional visuomotor metrics into our analysis: gaze-to-target distance (GTD), and gaze-to-user distance (GUD). These metrics represent the Euclidean distances from the current gaze position to the target and user positions, respectively. GTD measures the precision of visual tracking by indicating how closely participants’ gaze follows the target. However, GUD is a less informative and more delicate metric to handle: while it can reflect the degree of self-monitoring during the task, it may also covary with IDD simply because the user is closer to or farther from the target.

### Statistical analysis

We employed descriptive and inferential statistics of directional data [38]. We transformed the trajectory directions into unit vectors to calculate the average directional angles and computed the mean resultant vector to extract the mean orientation. We utilized the Rayleigh test to assess departures from circular uniformity (for data exhibiting a unimodal distribution). We used the Watson-Williams test to evaluate whether the mean directions of two or more experimental conditions were identical. This test is a circular analog of the one-factor ANOVA and assumes underlying von Mises distribution with equal concentration parameter [38]. We used a multi-sample non-parametric test to compare multiple samples to explore for equality of means or medians across conditions (circular analog to the Kruskal-Wallis test). We used multivariate analysis of variance (MANOVA) to test whether experimental conditions had the same mean. We also used linear regression models to evaluate whether predictors explained the variability in the dependent variable and employed ANOVA tests to assess the overall significance of the models. We employed the Friedman test as a non-parametric alternative to the repeated-measures ANOVA, to assess the equality of medians across several repeated-measures univariate groups. We illustrate our data as mean values ± standard error of the mean (S.E.M.) and consider statistical significance at a threshold of *P* ≤ 0.05. We report the test statistic (*F*-statistic along with the degrees of freedom for the model and residuals for ANOVA or χ^2^ for Friedman tests), and the *P*-value for the main effect for analyzed factors. We applied Bonferroni corrections to control for Type I errors following some tests. We indicate the number of participants included in the analysis for each task within the corresponding figure panels.

## Results

### Manual pursuit and interception behavior during chasing trials

**Fig 1:**
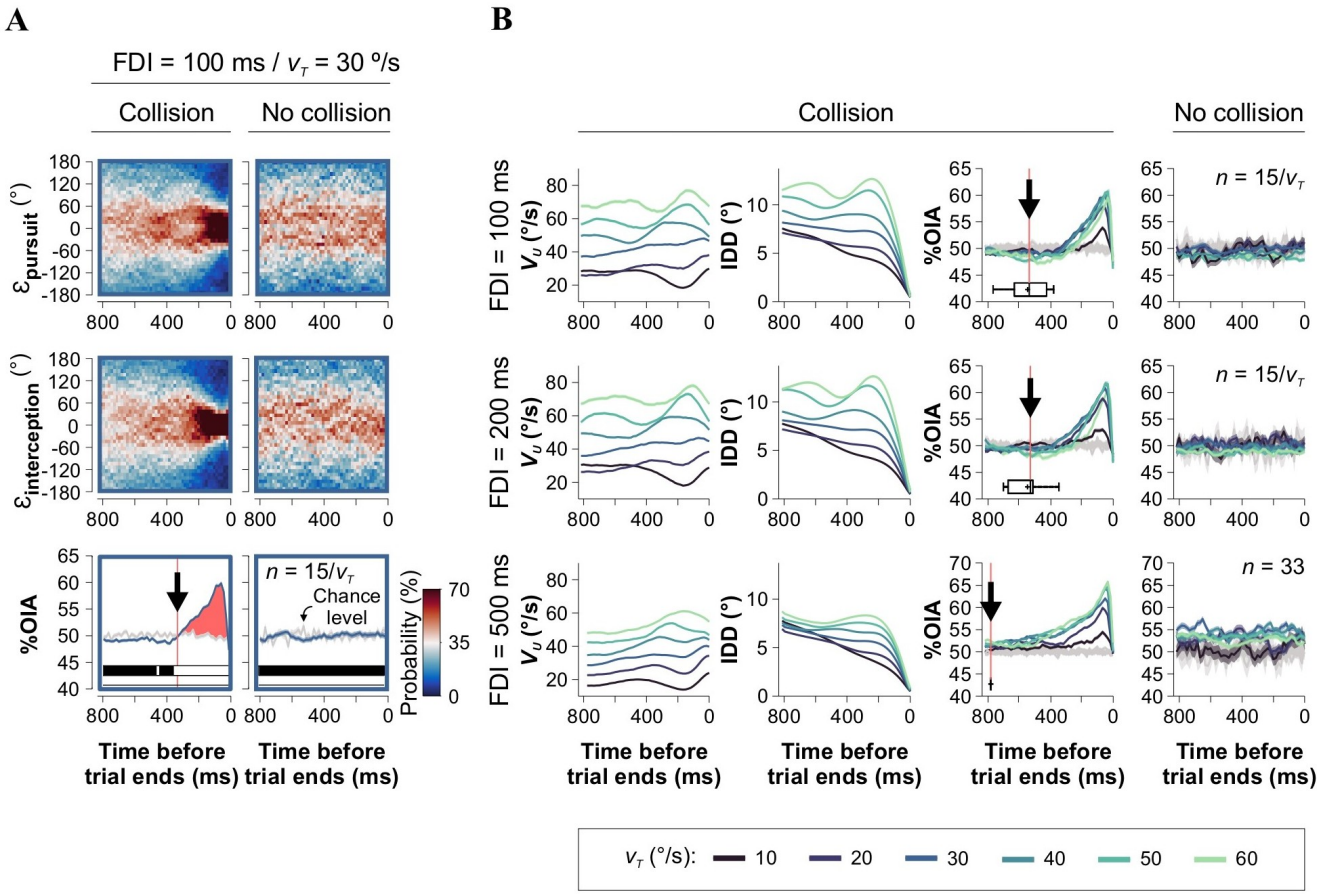
Virtual task to study chasing behavior. **(A)** Probability distributions of manual pursuit and interception errors for trials with (left column) and without (right column) collision. Traces below show corresponding % Optimal interception angle (%OIA) traces. Statistical tests below the panel are used to detect the onset of the %OIA trace. **(B)** Collision-triggered group averages of user speed (*v*_*U*_), inter-dot distance (IDD), and %OIA as a function of *v*_*T*_ (depicted in the color bar at the bottom). Panels arranged from lowest to highest FDI suggest that participants initiated the interception strategy sooner with higher FDI values. Panels to the right depict the average %OIA from trials with no collision.

We employed a simple visuomotor task in which participants used a joystick to interact with a computer-controlled dot moving along two-dimensional trajectories (S1 Fig in S1 File). This virtual setup allowed us to manipulate the speed of the target (*v*_*T*_) and the characteristics of its movement, including the magnitude (AR) and frequency (FDI) of directional changes (see Methods). Using data collected from participants performing this task, we first quantified the Optimal Interception Angle (%OIA, [29]), which reflects the degree of interception strategy employed on each frame. %OIA values range from 0 to 1, where 0 represents simple manual pursuit, 1 represents pure interception, and values above 50% indicate a tendency toward the interception strategy in the observed trajectories. Next, we analyzed the dynamics of angular errors from these distributions as participants approached the target, explicitly investigating whether there was a gradual tendency to produce smaller angular errors (*i*.*e*., an increase in precision before collision). We calculated the probability distributions of ε_pursuit_ and ε_interception_ over time, referenced to the moment when the trials ended. We illustrate these distributions as colormaps in Fig 1A. As expected, for collision trials, the probability distributions converged towards a mean zero error as the user approached the target (a 0 error denotes optimal pursuit or interception angle, respectively). However, this trend was strongly reduced in trials without collision (right panels in Fig 1A). Therefore, the %OIA progressively increased as effective collision approached. In contrast, %OIA did not exhibit such an increase in trials without collision (lower-right panel in Fig 1A), or in trials with simulated randomized directions (%OIA_rnd_, gray traces in the lower panels of Fig 1A).

We utilized the %OIA to distinguish between manual pursuit and interception behaviors, and to detect frames when participants employed the interception strategy. A %OIA < 50% suggests a preference for a manual pursuit strategy, as participants directed the joystick toward the moving target. On the other hand, a %OIA > 50% indicates a tendency towards an interception trajectory, with participants aiming the joystick towards the expected future position of the moving target. As before, the group averaged %OIA traces revealed that participants progressively increased their use of the interception strategy (*i*.*e*., %OIA > 50%) as they neared the target, especially towards the end of the chasing trial (lower-left panel in Fig 1A). We employed two-sample Kolmogorov-Smirnov (KS) tests across temporal frames to compare: i) the ε_pursuit_ *vs*. ε_interception_, and ii) the %OIA *vs*.%OIA_rnd_ distributions. By adopting a 5% significance level, we defined the onset of the interception strategy when both tests showed differences for two consecutive frames. This approach allowed us to detect the moment when participants started exhibiting interception behavior (for example, the black arrow in the left lower panel from Fig 1A is at 341.9 ms before collision). By differentiating pursuit-dominated from interception-dominated chasing frames, this method constitutes a valuable tool for studying the adoption of interception strategies.

Next, searching for conditions that could improve interception, we investigated the influence of task parameters, such as *v*_*T*_ and FDI, on the peak amplitude of the %OIA trace. We extracted data from participants solving the task at different FDI values (100 ms, 200 ms, or 500 ms), while *v*_*T*_ varied across a range of speeds (from 10°/s to 60°/s) in a randomly permuted fashion across trials (data from E_1_ and E_2_, refer to Table 1 for additional details regarding the experiments). We focused on examining the effects (and evolution) of three dependent variables: user speed (*v*_*U*_), inter-dot distance (IDD), and %OIA, during the final ~800 ms leading up to the collision. The *v*_*U*_ traces were proportional to the *v*_*T*_ values employed and were relatively stable throughout the analyzed time window leading up to the collision (first column in Fig 1B, [29]). The IDD traces were also scaled by *v*_*T*_, but decreased as the collision time approached and the participant neared the target (panels in the second column). Lastly, the %OIA traces showed a ~20% increase just before the collision (comparison between same temporal frames as in S1D Fig in S1 File; FDI = 100, 200, 500 ms, change in %OIA from 50.94% ± 0.13% to 61.23% ± 0.18%; Kruskal-Wallis test, with Mann-Whitney *post hoc* test, *F*_2,2241_ = 899.03, *P* < 0.001, panels in the third column; participants from E_1_ and E_2_), but such an increase was absent in trials where no collision occurred (change in %OIA from 50.06% ± 0.14% to 49.97% ± 0.14%, *F*_2,2133_ = 0.9759, *P* = 0.32; panels in the fourth column in Fig 1B). Furthermore, the variable onsets of the interception strategy (~539 ms, ~532 ms, and ~786 ms before collision, indicated by black arrows in panels from the third column in Fig 1B) suggests that participants initiated the interception strategy earlier with higher FDI values, in agreement with the target’s more predictable motion.

To examine %OIA in relation to space instead of time, we sorted the collision-triggered %OIA based on the distance to the target (IDD) within a time window of 25 frames (≈ 416 ms) before the collision. This approach ensured that parameters were arranged and averaged relative to IDD within the pre-collision time window. Once again, as participants neared the target, the sorted %OIA increased by ~10% (*F*_2,1107_ = 351.80, *P* < 0.001), but this increase was absent in trials without collision (*F*_2,1056_ = 0.05, *P* = 0.80; not illustrated). This enabled us to corroborate the observed increments in %OIA both temporally and spatially as participants approached the collision point. In our **Supplementary Material**, readers can access S1-S3 Tables in S1 File containing probabilities of successful interception across all examined experiments. Additionally, a dedicated section on ‘Task parameters that regulate interception behavior’ provides detailed insights into how various task factors influenced %OIA in both collision and no-collision trials.

### Gaze predictors of efficient interception

**Fig 2:**
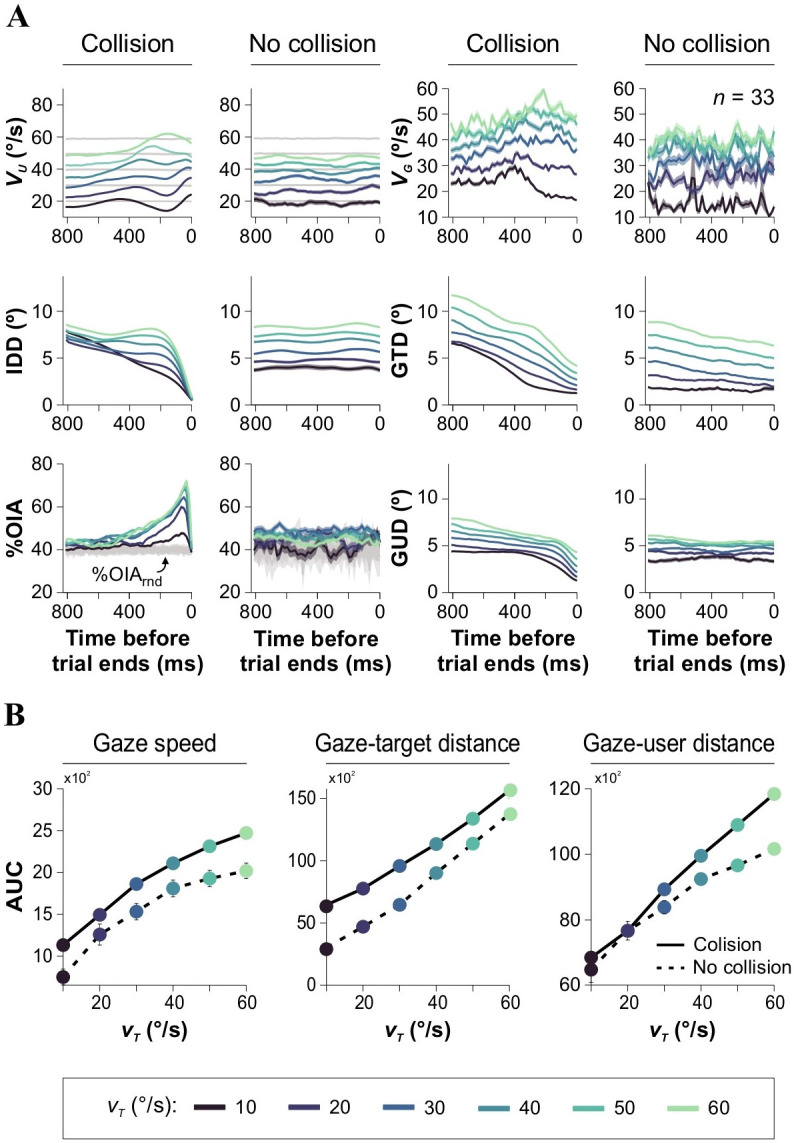
Oculomotor predictors of efficient interception. **(A)** Group averaged traces for user speed (*v*_*U*_), inter-dot distance (IDD), %OIA, gaze speed (*v*_*G*_), ‘gaze-target distance’ (GTD), and ‘gaze-user distance’ (GUD) as a function of *v*_*T*_. Note the differences between trials that lead to collisions and no collisions (labels on top of the panels). **(B)** Group averaged parameters extracted from the eye-tracking system. Increased *v*_*T*_ leads to increased gaze speeds, GTD, and GUD.

Although prior studies have suggested a connection between interception strategies and smooth pursuit eye movements [19], the exact relationship between gaze behavior and these strategies remains unclear. We used an eye-tracking system to investigate the impact of *v*_*T*_ on oculomotor responses during chasing trials (data from E_2_, FDI = 500 ms; calibration routines exemplified in S4 Fig in S1 File). As additional visuomotor metrics, we assessed the ‘gaze-speed’ (*v*_*G*_), the ‘gaze-target distance’ (GTD), and the ‘gaze-user distance’ (GUD), representing the Euclidean distances between the gaze and the target’s or the user’s positions, respectively. These metrics allowed us to analyze the participant’s proximity to the target. Fig 2A illustrates how *v*_*T*_ exerted control over motor and visuomotor performance, while the collision outcome also determined the observed behavioral responses. Regardless of the collision outcome, all visuomotor metrics increased with *v*_*T*_, indicating a consistent effect of target velocity on the participants’ visuomotor performance (Kruskal-Wallis test, with Mann-Whitney *post hoc* test, *v*_*G*collision_: *F*_1,4631_ = 147.34, *P* < 0.0001; *v*_*G*no-collision_: *F*_1,4631_ = 46.85, *P* < 0.002; GTD_collision_: *F*_1,4631_ = 587.08, *P* < 0.0001; GTD_no-collision_: *F*_1,4631_ = 649.81, *P* < 0.002; GUD_collision_: *F*_1,4631_ = 1565.1, *P* < 0.0001; GUD_no-collision_: *F*_1,4631_ = 1330.50, *P* < 0.002, *n* = 33; Fig 2B).

**Fig 3:**
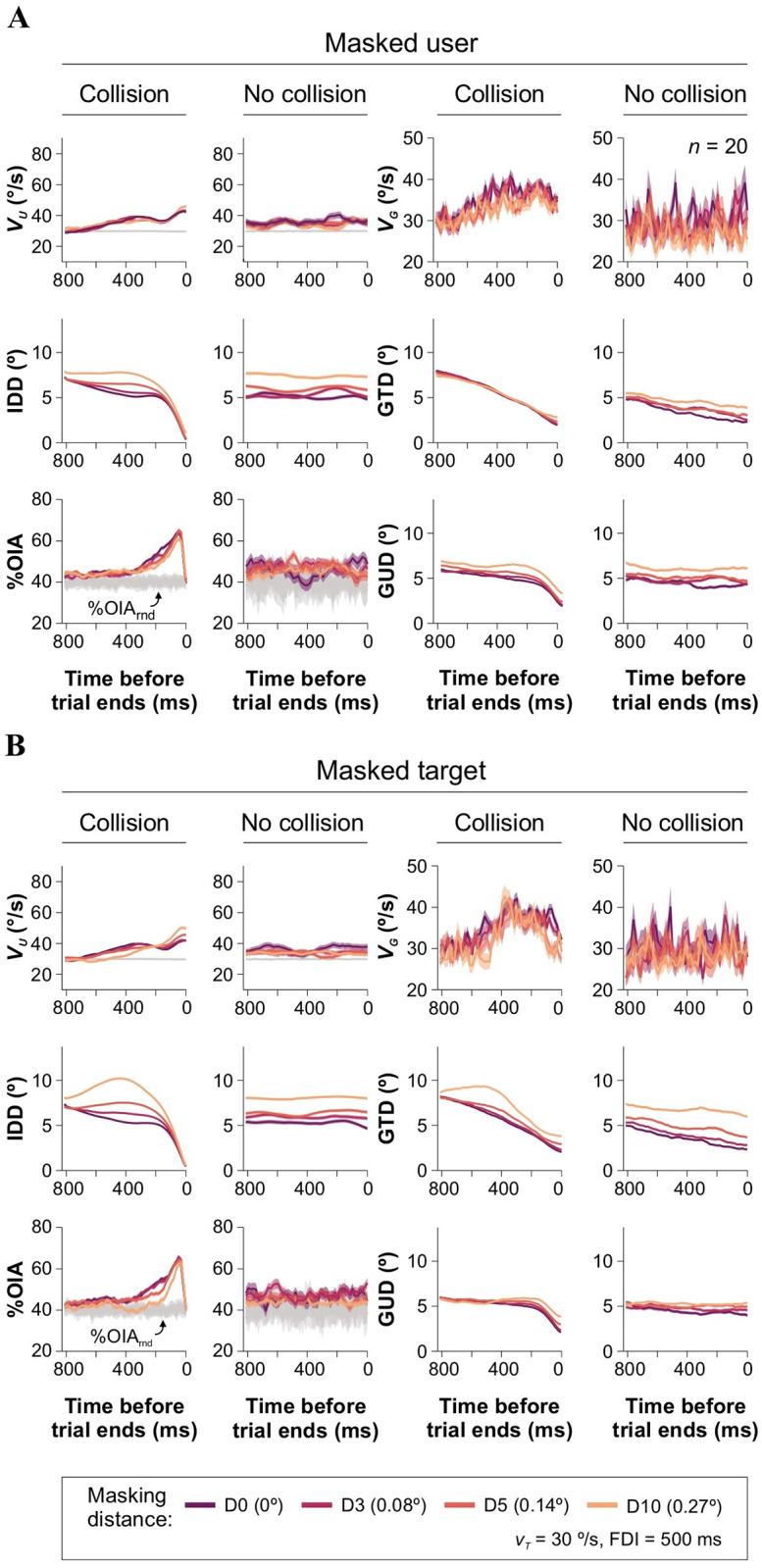
Visuomotor performance during spatial masking experiments. Panels (arranged as in previous figure) show group averaged traces for user speed (*v*_*U*_), inter-dot distance (IDD), %OIA, gaze speed (*v*_*G*_), ‘gaze-target distance’ (GTD), and ‘gaze-user distance’ (GUD) as a function of *v*_*T*_ for experiments where the user **(A)** or the target **(B)** were masked. Note that masking the user does not influence the GTD, whereas masking the target does impact the GTD. Gaze speed is not affected by masking distance. Experiments were performed at constant *v*_*T*_ = 30°/s, and FDI = 500 ms.

Next, we searched for changes in oculomotor responses associated with spatial occlusion experiments. We reasoned that masking should affect visualization and interception. Notably, we found that masking the user had minimal impact on the GTD (collision trials; data from E_3_ illustrated in Fig 3A). However, masking the target impaired the participants’ ability to approach the target efficiently, and the GTD increased with the target’s masking distance (Friedman test with two factors: masking distance, and masking condition [*i*.*e*., masked participant or target], with Bonferroni correction, χ^2^(2) = 813.75, *P* < 0.001 for all cases, *n* = 20; Fig 3B). As expected, participants showed reduced accuracy in approximating the target in trials without collisions, as evidenced by the IDD traces. However, despite IDD not showing a decrease over time, the GTD traces consistently displayed a negative slope, indicating an attempt to intercept the target. This finding suggests that participants continuously adjusted their gaze behavior to aim at the target whenever it was visible, exhibiting varying levels of effectiveness.

### Pupillary responses linked to efficient interception

**Fig 4:**
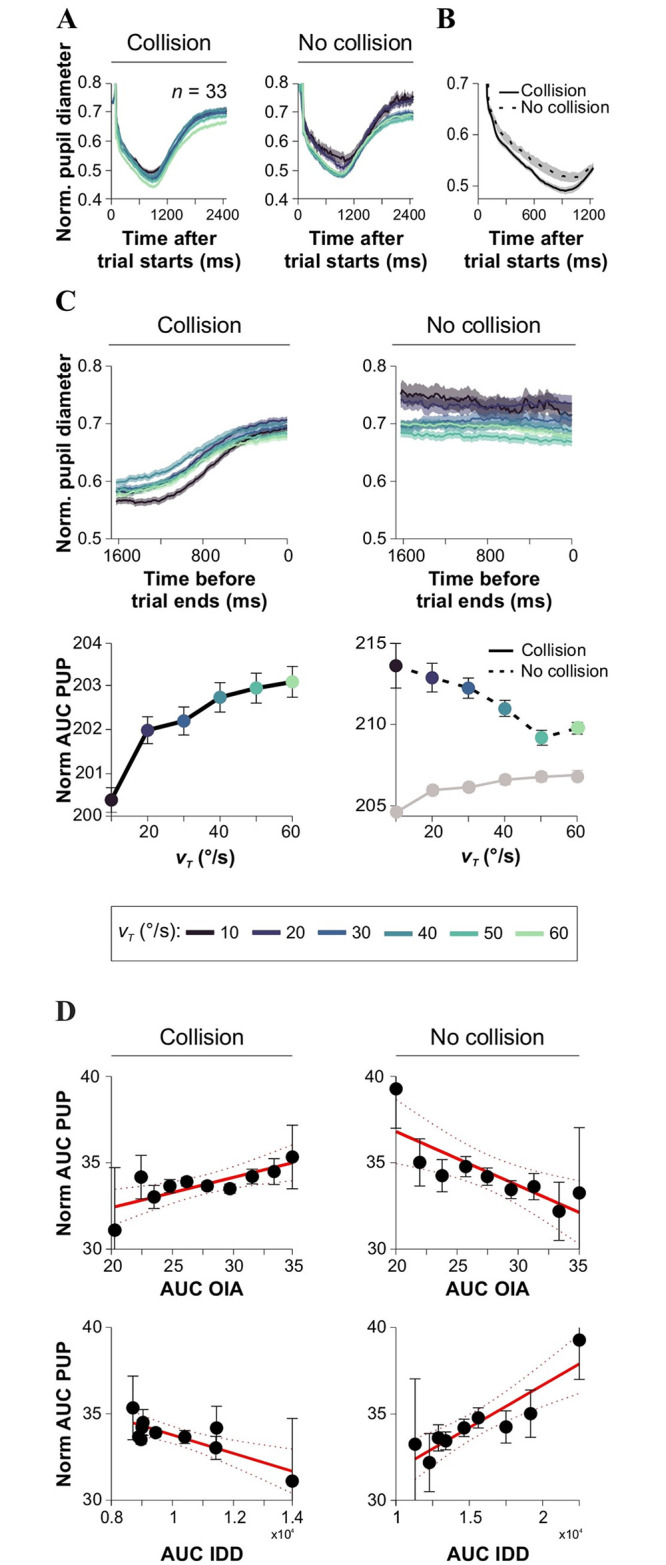
Pupillary responses predict efficient interception. **(A)** Normalized pupillary responses, aligned to the start of chasing trials, are illustrated for collision (left) and no-collision (right) trials, distinguished by various *v*_*T*_ values indicated by different colors. **(B)** Discernible differences between collision (black line) and no-collision (dotted line) trials became apparent around 300 ms after the start of the trial. **(C)** Normalized pupillary responses, aligned to the end of chasing trials, illustrated for collision (left) and no-collision (right) trials. Lower panels show the area under the curve of pupillary responses as a function of *v*_*T*_ for collision (left) and no-collision (right) trials. Gray dots represent the averaged values from collision trials (for comparison on the same y-scale). **(D)** Normalized pupillary responses against AUC %OIA (top) and AUC IDD (bottom) for collision (left) and no-collision (right) trials.

While joystick and eye movement data offer valuable information about an individual’s actions and gaze orientation, pupil data serves as an additional window into their cognitive state. For example, pupil dilation data can reveal the subjective difficulty of cognitive tasks, their intensity, and the underlying deployment of visual attention [25, 39]. Moreover, researchers have suggested that there is an increased cognitive effort with uncertainty processing, leading to larger pupillary diameters [39–41]. We investigated the influence of *v*_*T*_ and collision outcome on pupillary responses from participants solving our task (data from E_2_). We observed that the pupil contracted during the first ~50 frames ≈ 800 ms of the trial and dilated after that, showing similar patterns for collision and no-collision trials (Fig 4A). However, dilation amplitudes showed differences between collision and no-collision trials, consistent with a different build-up of decision uncertainty. More specifically, pupil responses were smaller for trials resulting in collisions (average diameter: 63.44% ± 0.33%, 5820 trials) but larger for trials without collisions (69.82% ± 0.38%, 4632 trials). Notably, these differences in pupil response diameters between collision and no-collision trials emerged approximately ~35 frames ≈ 730 ms after the beginning of the trial (Friedman test with *v*_*T*_ and collision outcome as factors, and with a Bonferroni correction applied, χ^2^ (2) = 1574.8, *P* < 0.001, *n* = 33; right panel in Fig 4B).

Subsequently, we investigated whether the pupillary differences linked to collision outcome were also present in the averaged traces aligned to the moment of collision (or to the end of the trial for cases with no collision). Pupillary responses increased as participants approached the moment of collision, whereas trials with no collision exhibited already elevated pupillary diameters that remained stable towards the end of the trial (Fig 4C). Interestingly, these pupillary responses were proportional to *v*_*T*_ for trials with collision but negatively proportional to *v*_*T*_ for trials without collision (slope of linear regression model, AUC PUP *vs*. *v*_*T*_; collision: ANOVA test, *F*_1,4_ = 18.94, *m* = 0.06, *P* = 0.01; no collision: *F*_1,4_ = 42.69, *m* = -0.14, *P* = 0.002; lower panels in Fig 4C). Furthermore, in trials with successful collisions, the AUC of pupillary responses (AUC PUP) increased with the AUC %OIA (slope of linear regression model, *F*_1,8_ = 11.41, *m* = 0.18, *P* = 0.009), but decreased with the AUC of IDD (slope of linear regression model, *F*_1,8_ = 15.84, *m* = -5.29⋅10^−4^, *P* = 0.004). For trials without collision, AUC PUP decreased with AUC %OIA (slope of linear regression model, *F*_1,8_ = 12.85, *m* = -0.28, *P* = 0.008) but increased with AUC IDD (slope of linear regression model, *m* = 5.05⋅10^−4^, *F*_1,8_ = 28.28, *P* = 0.001; Fig 4D). Therefore, there was a positive correlation between pupil dilation and *v*_*T*_ /%OIA for collision trials but a negative correlation for trials without collision.

**Fig 5:**
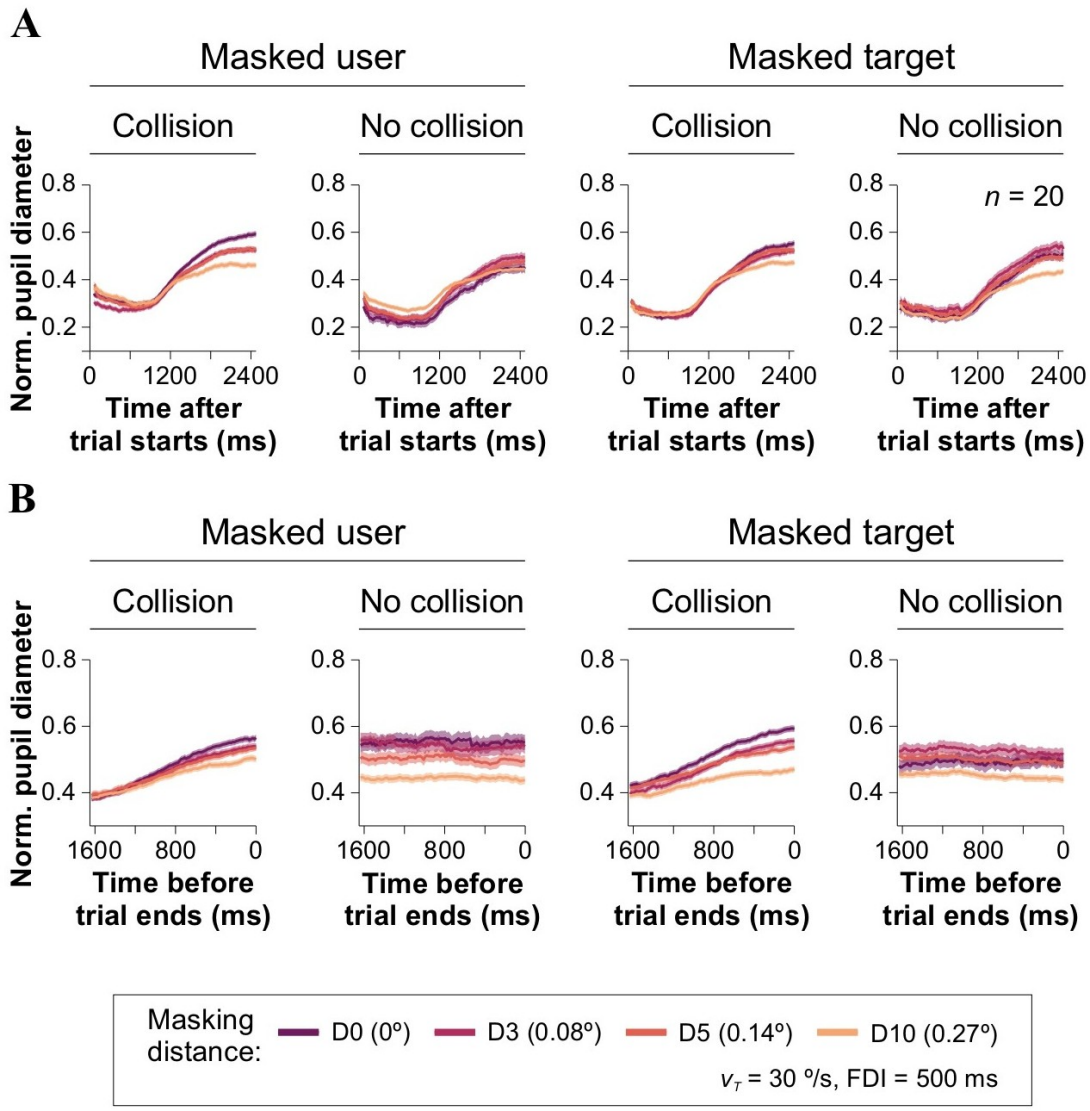
Masking distance decreases pupillary responses. **(A)** Normalized pupillary responses, aligned to the start of chasing trials, when masking the user (left) or the target (right). **(B)** Normalized pupillary responses, aligned to the end of chasing trials, when masking the user (left) or the target (right).

Using data from spatial occlusion experiments, we next analyzed the impact of the masking condition, masking distance, and collision outcome on pupillary responses (data from E_3_, *v*_*T*_ = 30°/s, FDI = 500 ms; Fig 5). Pupillary responses aligned to the beginning of the trials exhibited a similar pattern as previously described: an initial contraction followed by subsequent dilation for collision (average diameter: 47.26% ± 0.36%, 4720 trials) and no-collision (51.65% ± 0.43%, 3765 trials) trials (Fig 5A). When the target was masked, pupil responses were smaller for trials resulting in collisions, while they were larger for trials with no collisions. However, when masking the user, the trend reversed, with greater pupillary responses for collision than for no-collision trials. When analyzing traces aligned to the moment of collision, we observed, again, a similar trend as described earlier: pupillary responses increased as participants approached the collision moment, while trials without collisions consistently displayed elevated pupillary responses. Notably, when masking the user or the target, pupillary responses were reduced by increasing masking distance (Kruskal-Wallis test, with Mann-Whitney *post hoc* test, *F*_3,115697_ = 2.392⋅10^4^, *P* < 0.001, *n* = 20; Fig 5B). Therefore, in experiments with variable *v*_*T*_, pupil responses were smaller for collision trials and larger for no-collision trials (Fig 4C). In contrast, in spatial occlusion experiments, pupil responses were larger for collision trials compared to no-collision trials, and they were reduced by increasing masking distance (Fig 5B). The different observations in pupil responses between collision and no-collision trials and the masking condition could be attributed to the different cognitive demands and processing involved in each experimental condition.

**Fig 6:**
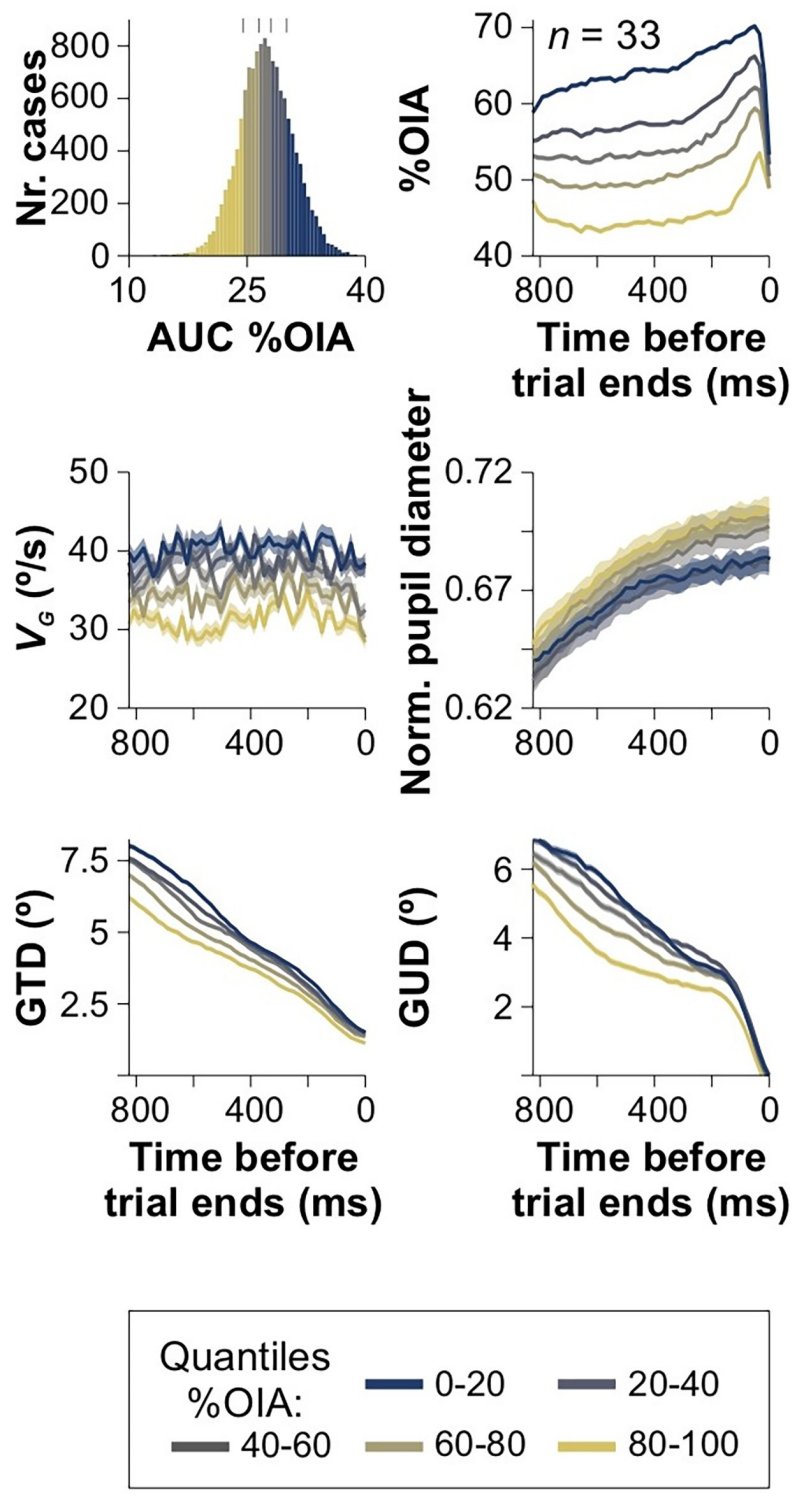
Distinct interception behavior patterns among graded interceptors. The distribution of AUC %OIA cases is divided into five subgroups based on ascending quantile ranges, color-coded in the accompanying colorbar. The gray tick marks above the distribution denote the start and end points for each category within each group. Each subgroup exhibits a unique profile in %OIA, gaze speed (*v*_*G*_), normalized pupillary diameter, ‘gaze-target distance’ (GTD), and ‘gaze-user distance’ (GUD). Detectable variations were observed across groups in %OIA, *v*_*G*_, and GTD.

To investigate the utilization of visual cues and gaze behavior during interception, we used AUC %OIA from individual traces to generate ranges of varying interception proficiency levels among trials, enabling us to detect potential patterns associated with successful interception. Using data from 13,449 collision trials (from E_2_), we established these ranges and categorized trials into five subgroups based on quantiles from the overall %OIA distribution (upper left panel in Fig 6). We then averaged all attributes from those traces for each participant and calculated the group-averaged traces for each attribute and each quantile category. By tagging the data from all these trials, we explored and compared the average visuomotor collision-triggered responses across categories. We employed a gradual color transition to represent each range, illustrating the different patterns of interception behavior among the various AUC %OIA categories (Fig 6). Notably, there were detectable differences across groups in %OIA, gaze speed, and GTD (Kruskal-Wallis test, with Mann-Whitney *post hoc* test, *F*_1,709_ ≥ 12.76, *P* ≤ 0.01 for all cases), but not so in pupillary responses and GUD (*F*_1,709_ ≤ 0.38, *P* ≥ 0.06, *n* = 33). These results reveal important interdependencies among %OIA, gaze speed, and GTD, suggesting that various aspects of interception skills are closely linked and could contribute to the overall performance in the task.

## Discussion

Chasing moving objects can involve a combination of pursuit and interception strategies. In joystick-based tasks, manual pursuit entails aiming directly at the target’s current position [11], while interception requires predicting the future location of the target and adjusting movements accordingly to reach the interception point [14]. Several elements, including target proximity, predictability of the target’s movement, speed, and external conditions, impact the selection between manual pursuit or interception. For instance, pursuing the target can be effective, especially when the target moves slowly and maintains a constant direction. However, in conditions with more unpredictability, a combination of predictive and prospective control can lead to more successful outcomes [42, 43]. Predictive control requires knowing the physical laws governing the target’s motion and the ability to make accurate predictions about future events [44, 45]. This approach involves estimating the contact point and time of the collision, enabling individuals to anticipate the trajectory of the target and execute their actions accordingly. However, a purely predictive control strategy also implies that adjustments to movement should not occur late in the execution, as the plan is well-established in advance [12, 14]. In contrast, prospective control does not explicitly predict the final collision point; instead, it relies on information about the future interception under the current conditions. Thus, prospective strategies enable continuous movement adjustments to achieve successful interception [9–11, 14, 46].

By calculating the optimal interception angle (%OIA, see Methods), we identified a time window within chasing trials when participants employed an interception strategy. To determine the onset and duration of interception, we developed a method that tracked the %OIA over time based on participants’ joystick behavior. Indeed, as participants approached the target, their %OIA increased in collision trials, especially with smaller target speed (*v*_*T*_) values, while this increase was absent in trials without collision. We hypothesized that task parameters like *v*_*T*_ and the fixed-direction interval (FDI, see Methods) influenced gaze behavior and interceptive performance. Our findings revealed a U-shaped relationship between the area under the curve (AUC) of the %OIA and *v*_*T*_, indicating that the interception strategy was most effective at intermediate target speeds. Furthermore, increasing the FDI led to higher AUC %OIA values. These longer FDI likely gave participants more exposure to the target’s movement direction, facilitating better adaptation to its motion dynamics. Probably, such extended exposure allowed participants to adjust their chasing behavior strategically, resulting in an enhanced ability to predict the optimal interception angle and, therefore, increased their %OIA. In our spatial occlusion experiments, we masked the user or the target during the interception phase. The absence of these visual cues impeded participants’ ability to assess and adjust their chasing orientation angles, leading to a relative decrease in %OIA. These findings highlight a robust bidirectional influence of task parameters on the interception strategy, contingent on the amount of available information. We have shown the relationship between successful target interception and experimental parameters, such as *v*_*T*_, FDI, and masking distance, in a previous publication [29]. These findings contribute to the interpretation of comparisons between collision and non-collision trials.

We reasoned that ocular movements should precede and inform joystick control during the interception task. We developed a method to detect the onset and duration of significant changes in %OIA and combined it with eye-tracking recordings to investigate the visuomotor predictors of interception. We analyzed participants’ eye movements and explored the interrelationship between interception and visuomotor performance. As *v*_*T*_ increased, we observed proportional effects on gaze speed (*v*_*G*_), gaze-to-target distance (GTD), and gaze-to-user distance (GUD, see Methods). This increase in *v*_*T*_ forced participants to adjust their *v*_*G*_ to cover larger distances between the target and the user, thereby enhancing their ability to effectively intercept the moving target. Additionally, when masking the user, the main impact was an increase in GUD, whereas masking the target resulted in a rise in GTD. These results confirm that eye movements were closely linked to interception performance, further suggesting that interception strategies were optimized to match (and constrained by) the proficiency of the ocular pursuit system [19]. Humans exhibit anticipatory eye movements toward the future positions of moving objects, even without intending to intercept them. As eyes typically lead hand movements, it’s reasonable that GTD was smaller than the inter-dot-distance (IDD, see Methods) before interception [15–18, 22]. However, larger GTD values don’t necessarily indicate poorer performance. Quite the opposite: they may reflect larger predictions of future positions. Indeed, in our case, the larger GTD values suggest stronger predictions for collision trials compared to no-collision trials. Additional metrics, including initial saccade latency, the precision of predictive saccades, pursuit gain, and tracking errors, among others, have also served as indirect indicators of prediction processes [47–50]. Even in the simplest manual pursuit tasks, predictive processes are likely involved due to sensorimotor processing delays, necessitating prediction for successful interception [20, 51].

When analyzed by percentiles, the strong relationship between %OIA and gaze-target distances suggests a potential influence of eye movements on interception %OIA. These findings reveal the integrated role of oculomotor behavior in visual perception and motor control during our interception task. Some critics argue that the computational demands linked to internal representations for planning and executing movements could limit flexibility across different tasks. To address this, continuous online monitoring of movement properties, such as trajectory and velocity, becomes essential for future control planning [14]. From this perspective, the interception task is transformed into a dynamic process, with the timing and location of interception gradually emerging as the movement unfolds [52]. The main benefit of this approach is that it ‘liberates’ performers from relying solely on single cues and allows them to adapt and respond to unexpected changes. Numerous studies support the notion that interception involves real-time decision-making, which can lead to increased errors under challenging conditions [9, 10, 14, 46, 53].

The average human pupil size is around 3 mm, but it can vary between 2 to 8 mm when exposed to different light intensities [54]. In fixed luminance conditions, however, pupillary changes are typically much more modest than those observed in response to light, rarely exceeding 0.5 mm. Believed to reflect attentional processes, these changes in pupil diameter occur binocularly and without conscious control [23]. Researchers have linked pupillary diameter changes to surprise processing, uncertainty [25], and decision bias-related variables [24]. A prevailing view suggests that the brain communicates uncertainty signals throughout neural circuits that span the entire brain via low-level arousal systems [55, 56]. Consequently, alterations in the global brain state caused by uncertainty may impact adjustments in decision-making. Interestingly, pupil size is coupled (at least to some degree) with the locus coeruleus (LC) activity, which serves as the central hub of the norepinephrine system [24, 55]. Studies have suggested that micro-stimulation of the LC or the superior and inferior colliculi induces pupil dilations, highlighting its role as an indirect measure of brain activity and arousal [57–59] (but see [24]). The experimental results from these previous studies illustrate the multifaceted nature of pupillary responses in capturing both cognitive and affective states.

Our results revealed that pupillary responses exhibited distinct patterns depending on the collision outcome. The pupil diameter initially reduced in size but then increased for all chasing trials. Notably, pupil diameters were smaller for collision trials and larger for no-collision trials, supporting the notion that, in our task, pupillary diameter may be inversely related to decision uncertainty. More specifically, the larger pupil size observed in no collision trials may indicate that participants “gave up” or disengaged from the current trial as they became increasingly confident that they had failed to intercept the target. We also found that the extent of pupil dilation was positively correlated with *v*_*T*_ and the AUC %OIA and negatively correlated with the AUC IDD for collision trials. Pupil dilation showed a negative correlation with *v*_*T*_ and AUC %OIA for trials with no collision but a positive correlation with AUC IDD. Interestingly, our spatial occlusion experiments revealed smaller pupillary responses in trials with larger masking distances. These findings suggest that uncertainty about the target’s and user’s location (due to increased masking distances) was associated with reduced pupillary responses during the interception task. Therefore, pupil dilation in our task was associated with task difficulty, providing insights into the dynamic relationship among visual attention, cognitive effort, and interception. Previous works have reported that pupil diameter is related to both response accuracy and the probability of correct choices [60]. In other tasks, early pupil dilation is larger when the value of the chosen option is more uncertain, while late pupil constriction is greater when the outcome violates the expected value, indicating that pupil responses could track surprise and feedback valence [61].

Various limitations in our study should be highlighted. Firstly, the eye tracker’s low sampling frequency limits reporting on saccades, potentially impacting the interpretation of dynamic eye movements in our study. Another critical factor arises from the implicit human visuomotor delay, which typically ranges from 100–200 ms for continuous target motion and 200–300 ms for abrupt changes. Therefore, our experimental design, using FDI values of 100 and 200 ms (data from E_1_), may not adequately capture stimulus-induced changes in movement direction. Regarding pupillary responses, one possibility is that the observed differences in pupillary diameter could be attributed to variations in luminance. Participants who efficiently kept the target (black dot) closer to the fovea might have experienced reduced net luminance reaching the eye compared to those who focused or foveated more on the background. However, this explanation seems unlikely. Given that pursuit gain is higher at slower temporal frequencies [62], we would expect pursuit efficiency to be negatively related to *v*_*T*_. Yet, we observed greater pupillary diameters with higher *v*_*T*_. Another concern is that the difference in trial durations between collision and no-collision trials could introduce a potentially confounding factor when comparing pupillary traces. This highlights the need for careful experimental design to ensure a balanced comparison. Therefore, it is essential to account for these and other confounding variables that may impact pupillary changes in our interception task. Future experiments should better control these variables to enable a more thorough exploration of the relationship between pupil diameter and uncertainty [35]. Finally, we acknowledge our study’s limitation in external validity, and endorse the idea of integrating more ecologically valid tasks, such as those created in virtual reality environments [63–66].

Various algorithms can be employed to implement successful interception behavior [12, 14, 66, 67]. Studying the visuomotor predictors of interception is crucial for understanding how the human brain coordinates eye and hand movements for precise interception. This work offered analytic tools to investigate interception behaviors and their underlying mechanisms. Future studies could explore the impact of perceptual biases, assess gaze training effects, and explore cognitive mechanisms involved in interception strategies, advancing our understanding of human adaptability in dynamic environments. The relationship between %OIA and gaze behavior could inform training protocols and assistive technologies, benefiting fields like sports science, neuro-motor rehabilitation, and robotics. The study of sensorimotor integration could also have broader implications for cognitive abilities and academic performance [68].

Our study explored visuomotor predictors of interception, revealing how the target’s speed and predictability influenced manual pursuit and interception strategies. Through meticulous motor and visuomotor performance analysis, we found that participants consistently adapted their gaze behavior in collision trials, demonstrating their capacity to adjust to changes in the target’s direction throughout chasing trials. Pupillary responses were also influenced by task conditions and collision outcomes, suggesting different levels of uncertainty processing. Additionally, the utilization of visual information varied, resulting in distinct patterns of interception performance. Our research contributes to understanding effective interception strategies and the intricate relationship between eye movements and interception performance.

## Supporting information

S1 File (PDF)
