# Supplementary material for: Visuomotor predictors of interception
Source: PLoS One. 2024 Sep 16;19(9):e0308642. doi: 10.1371/journal.pone.0308642 (PMC11404793; doi:10.1371/journal.pone.0308642)
Supplement: S1 File — (PDF) [file pone.0308642.s001.pdf]

Supplementary Material for

Visuomotor predictors of interception

Inmaculada Márquez[1,2], Mario Treviño[3*]

[1]Departamento de Ciencias Médicas y de la Vida, Centro Universitario de la Ciénega, Universidad de Guadalajara, Ocotlán, México
[2]Laboratorio de Conducta Animal, Departamento de Psicología, Centro Universitario de la Ciénega, Universidad de Guadalajara, Ocotlán, México
[3]Laboratorio de Plasticidad Cortical y Aprendizaje Perceptual, Instituto de Neurociencias, Universidad de Guadalajara, Guadalajara, Jalisco, México

*** Corresponding author:**

Dr. Mario Treviño (mariomtv@hotmail.com), Laboratorio de Plasticidad Cortical y Aprendizaje Perceptual, Instituto de Neurociencias, Universidad de Guadalajara. Francisco de Quevedo 180, Arcos Vallarta. C.P. 44130. Guadalajara, Jalisco, México.

Exp.	FDI (ms)	V_T (°/s)	Probability of interception			Number of participants
E_1	100	10	95.39%	±	1.36%	15
	200	10	97.19%	±	0.96%	
	100	20	86.75%	±	1.83%	15
	200	20	89.69%	±	1.21%	
	100	30	74.49%	±	2.28%	15
	200	30	76.01%	±	3.86%	
	100	40	64.23%	±	4.08%	15
	200	40	66.45%	±	4.15%	
	100	50	50.91%	±	2.59%	15
	200	50	54.05%	±	3.18%	
	100	60	43.87%	±	2.14%	15
	200	60	42.42%	±	2.92%	
E_2	500	10	96.76%	±	1.05%	33
	500	20	91.95%	±	1.41%	
	500	30	87.04%	±	1.68%	
	500	40	80.32%	±	2.18%	
	500	50	73.92%	±	2.19%	
	500	60	66.95%	±	2.05%	

Sup Table 1. Probability of successful interception (E_1, E_2). The table illustrates the group probability of interception for E_1 and E_2, as detailed in **Table 1**.

Exp.	FDI (ms)	V_T (°/s)	Masking distance (°)	Probability of interception			Number of participants
E_3	500	30	0	91.73%	±	1.44%	20
	500	30	0.08	88.34%	±	1.43%	
	500	30	0.14	83.99%	±	1.55%	
	500	30	0.27	70.74%	±	2.09%	
E_6	500	10	0	99.47%	±	0.11%	20
	500	10	0.08	97.24%	±	0.51%	
	500	10	0.14	93.37%	±	1.09%	
	500	10	0.27	85.36%	±	1.43%	

Sup Table 2. Probability of successful interception (E_3, E_6). Probability of successful interception. The table illustrates the group probability of interception for E_3 and E_6, as detailed in **Table 1**.

Exp.	FDI (ms)	V_T (°/s)	Probability of interception			Number of participants
E_4	100	10	98.34%	±	0.74%	20
	300	10	98.35%	±	0.69%	
	400	10	97.96%	±	0.87%	
	500	10	98.32%	±	0.70%	
	1000	10	98.48%	±	0.78%	
	1500	10	98.58%	±	0.67%	
E_5	100	30	89.18%	±	1.53%	40
	300	30	85.92%	±	1.52%	
	400	30	87.39%	±	1.48%	
	500	30	88.02%	±	1.55%	
	1000	30	88.46%	±	1.60%	
	1500	30	89.01%	±	1.50%	

Sup Table 3. Probability of successful interception (E_4, E_5). The table illustrates the group probability of interception for E_4 and E_5, as detailed in **Table 1**.

Task parameters that regulate interception behavior

To examine the influence of task parameters on the amount of interception strategy before collisions, we analyzed how they affected the area under the curve (AUC) of the %OIA trace. For collision trials, we found an inverted U-shaped relationship between AUC %OIA and v_T, indicating that the interception strategy peaked at intermediate v_T values (data from E_1 with FDI = 100 ms and 200 ms; left upper panel in **S2A Fig**). This implies that participants were more likely to adopt an interception trajectory when the target moved at moderate speeds. Interestingly, with an FDI of 500 ms, the AUC %OIA increased with v_T until it reached a plateau for values greater than 40°/s (left lower panel in **S2A Fig**). This analysis allowed us to assess the divergence between trials with and without collisions (averaged AUC data for no-collision trials in the right panels from **S2A Fig**). The results indicate that v_T, FDI, and collision outcome (presence or absence of collision) influenced the AUC %OIA (Kruskal-Wallis test, with Mann-Whitney *post hoc* test, $F_{3,1456} = 531.1$, $P < 0.0001$). We corroborated this effect using multivariate linear regression analysis with %OIA as the independent variable and v_T, FDI, and collision outcome as the dependent variables. The overall MANOVA test confirmed the significance of the multivariate regression (Rao's $F_{3,1456} = 364.4$, $P < 0.0001$; participants from E_1 and E_2 grouped). Next, we reasoned that a longer FDI could enhance %OIA values by allowing participants to assess the optimal interception angle better, refine interception strategies, and adapt to target motion dynamics. Thus, we investigated the impact of various FDI values on AUC %OIA (average traces from E_5 are illustrated in **S2B Fig**). We found that there was a sigmoidal increase in AUC %OIA as FDI increased, with peak values achieved at FDI ≥ 300 ms for both collision and non-collision trials (Kruskal-Wallis test, with Mann-Whitney *post hoc* test, $P < 0.0001$; MANOVA, $F_{3,719} = 43.54$, $P < 0.0001$, $n = 60$; data from E_4, with $v_T = 10°/s$, is illustrated in the upper panels, while data from E_5, with $v_T = 30°/s$, is presented in the lower panels of **S2C Fig**). These psychometric fits served to identify the critical FDI value at which the %OIA saturation occurred.

We hypothesized that masking either the user or the target could disrupt the availability of critical visual cues during the interception phase, decreasing accuracy in assessing and adjusting directional angles and thereby impacting the %OIA. Additionally, masking stimuli could alter attention allocation and divert cognitive resources away from interception processes, further influencing the %OIA. Using an *in situ* spatial occlusion technique [1], we conducted experiments with a new group of participants without prior experience with the task. We eliminated the participant's (*i.e.,* 'masked user') or the target's (*i.e.,* 'masked target') visibility at various inter-dot masking distances (0°, 0.08°, 0.14°, and 0.27° of visual angle; these values were the same across all participants) with a fixed $v_T = 30°/s$, and an

FDI = 500 ms (data from E_3). The experimental results demonstrate the effects of masking the user (**S3A Fig**) or the target (**S3B Fig**) under balanced conditions (balanced collision trials with masked user = 49.95% ± 1.52%). In agreement with our hypothesis, increasing the masking distances had a detrimental effect on the efficiency of the interception process, evidenced by larger IDD and smaller %OIA traces during the pre-collision time window. The detrimental effect was further supported by calculating the AUC %OIA for trials with (left panel) and without (right panel in **S3C Fig**) collision, highlighting the importance of maintaining clear visibility of both the participant and the target for successful interception (Friedman test with four factors: v_T, masking distance, masking condition [*i.e.*, masked participant or target], and collision outcome, with Bonferroni correction, $\chi^2(4) = 3143$, $P < 0.001$ for all cases with the exception of masking condition, $n = 40$). Notably, the masking distance did not change the AUC %OIA for trials with a collision at $v_T = 10$ °/s, and FDI = 500 ms (Friedman test with two factors: masking distance, masking condition [*i.e.*, masked participant or target], with Bonferroni correction, $\chi^2(2) = 203.2$, $P < 0.001$ for all cases, $n = 20$; data from E_6 illustrated in **S3D Fig**). This observation suggests that at lower v_T, participants relied less on visual cues for successful collisions. In other words, slower v_T could allow efficient collisions even when visual cues are masked at variable inter-dot distances. Therefore, at $v_T = 10$ °/s, the %OIA remained relatively unaffected by masking distance as participants effectively preserved their interception strategies.

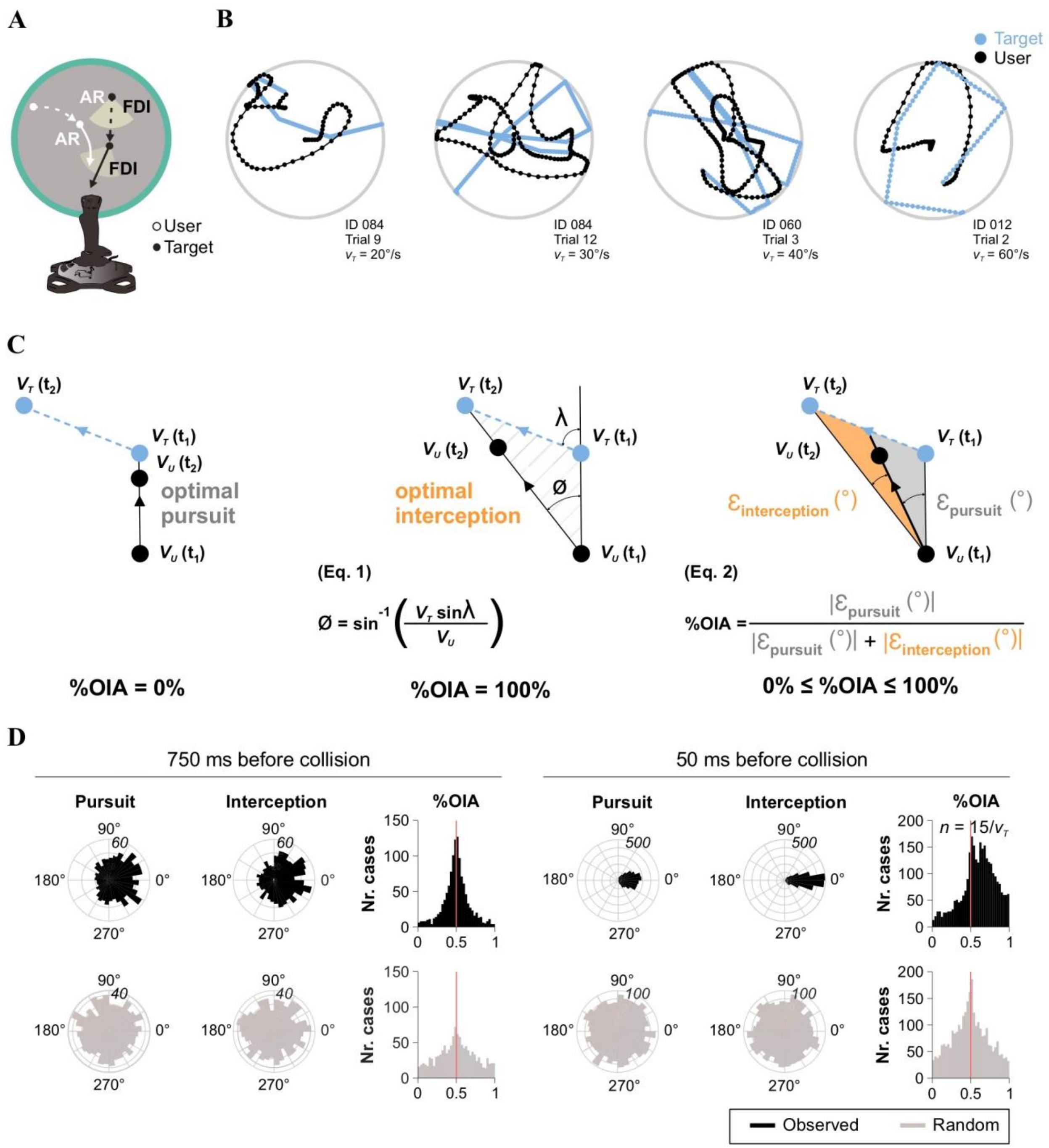

Sup Fig 1. Optimal strategies to intercept a target. (**A**) Cartoon of the experimental arrangement. Participants sat in a chair and used a joystick to control the movement of a white dot. The target, a black dot controlled by the computer, followed straight paths (straight black lines), and altered its course at regular intervals referred to as 'fixed-direction intervals' (FDI). The user's trajectory is illustrated by a white curved line. **(B)** Sample target (sky blue) and user (black) trajectories recorded during our experiments. Note how the target trajectories are rectilinear, whereas the user trajectories exhibit more curvature due to participants controlling the joystick. Dots on these trajectories indicate the frame captures. **(C)** The target (sky blue dot) moves in a straight line at a constant speed of v_T. The user (black dot) moves at an instantaneous speed of v_U. The straight vertical line depicts the optimal pursuit trajectory (panel on the left). The optimal interception trajectory, described by Eq. 1, represents the shortest intercept path achievable,

considering the speeds of both the user and the target (panel on the middle). For every acquired frame, the optimal pursuit and interception vectors were computed. Subsequently, the participant's observed direction was compared to these references, allowing for the calculation of angular errors. The $\varepsilon_{pursuit}$ represents the angular error between the observed trajectory and the optimal pursuit trajectory (gray shaded area), while the $\varepsilon_{interception}$ represents the angular error between the observed trajectory and the optimal interception trajectory (orange shaded area, panel on the right). **(D)** Polar panels show the distributions of angular manual pursuit and interception errors at two intervals before collision. Lower panels show the distributions obtained from randomly generated chasing directions. The max. range of the polar plots is shown in *italics*.

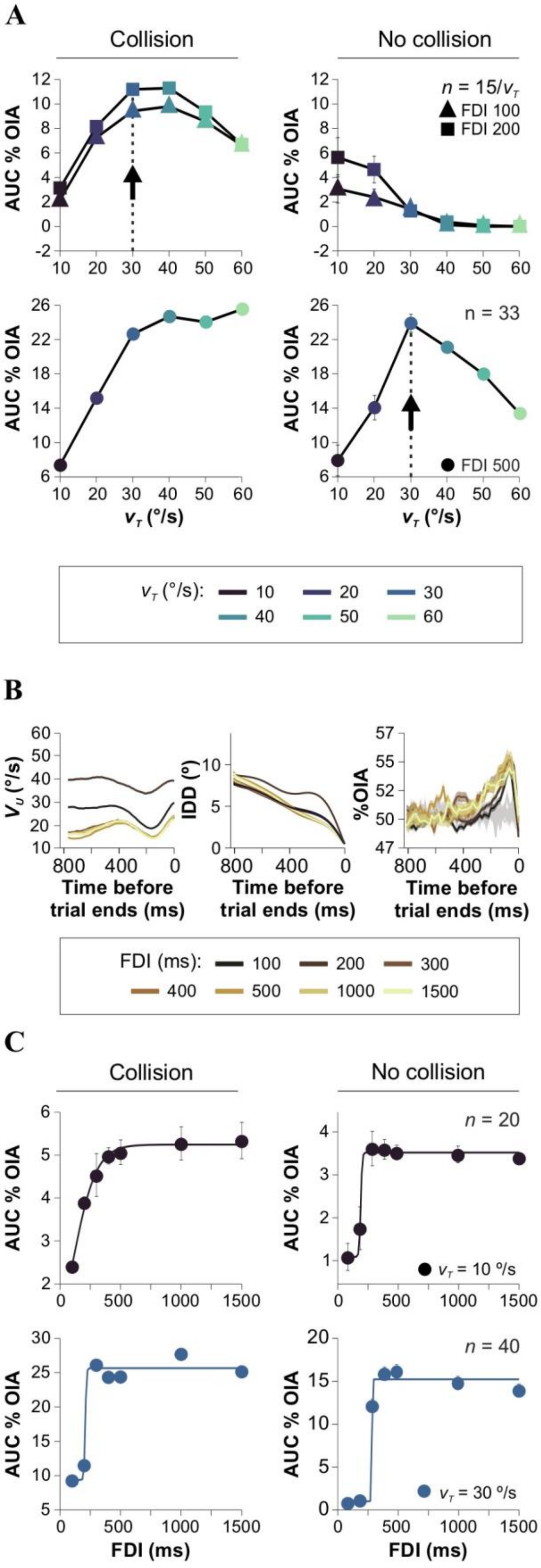

Sup Fig 2. Optimal conditions for interceptive behavior. (A) Area under %OIA trace as a function of v_T from trials with (left panels) and without (right panels) collision. v_T values depicted in the colorbar at the bottom. **(B)** Group averaged traces for user speed (v_U), inter-dot distance (IDD), and %OIA as a function of FDI, depicted in the colorbar at the bottom. **(C)** Area under averaged %OIA traces as a function of FDI for experiments with v_T = 10 °/s (upper panels in black), and v_T = 30 °/s (lower panels in kashmir blue). These figures reveal saturation of %OIA at FDI ≥ 500 ms. For this reason, in our main figures, we incorporated smaller, 'unsaturated' FDI values (100 ms, 200 ms) alongside 500 ms to explore the dynamics of the observed phenomena. The color coding of these plots aligns with the v_T colorbar used in the main figures. *Note*: Initially, our experimental design for E4 and E5 involved 20 participants per experiment, with random assignment until reaching this quota. However, the psychometric curve for experiments involving v_T = 30 °/s displayed a sharp transition with a very high slope. To ensure this was not an artifact due to a small sample size, we doubled the participants for E5 (see **Table 1**). Despite this increase, the psychometric curves retained their sharp transition.

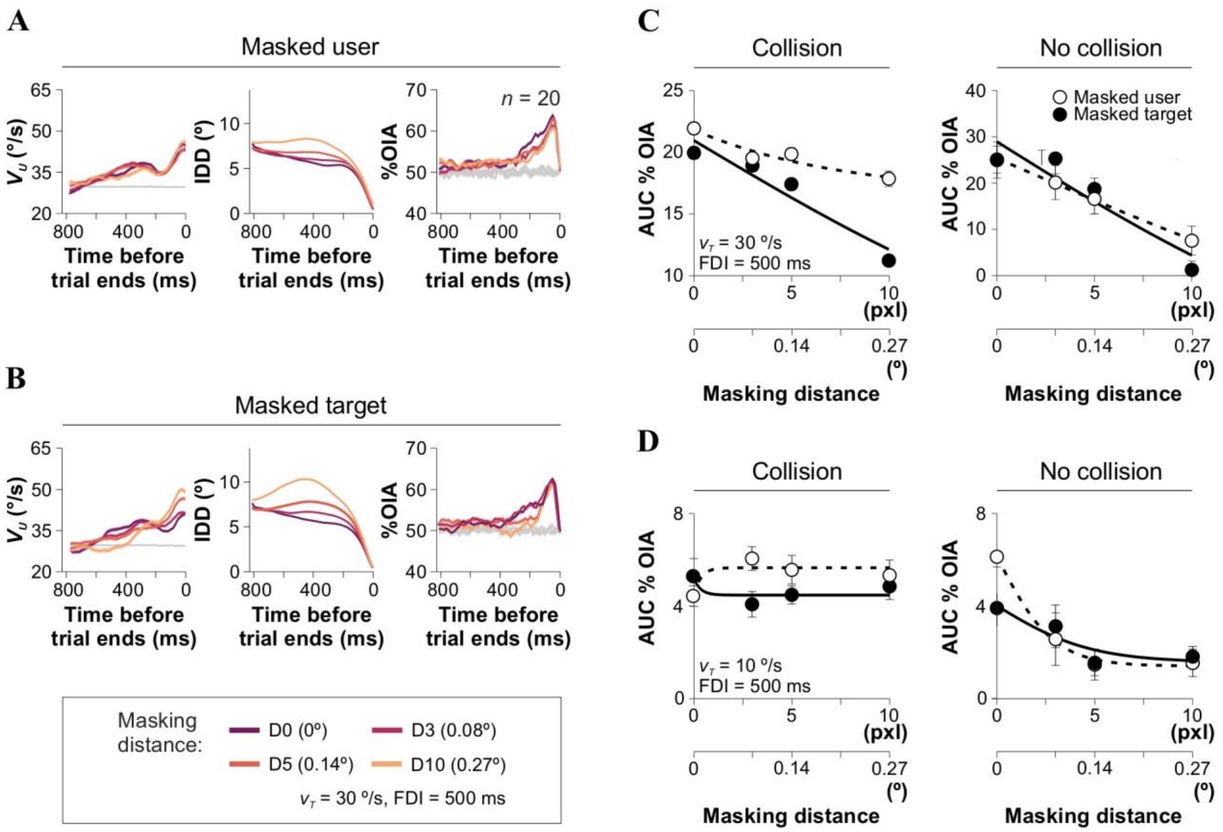

Sup Fig 3. Masking the user or the target reduces the efficiency of interceptive behavior. Masking the user or the target reduces the efficiency of interceptive behavior. Group averaged traces for user speed (v_U), inter-dot distance (IDD), and %OIA as a function of masking distance, depicted in the colorbar at the bottom. Masking the user (upper panels, **A**) or the target (lower panels, **B**) increases v_U and IDD. **(C)** Area under averaged %OIA traces as a function of masking distance ('masked user' in empty circles, 'masked target' in black) for experiments performed with v_T = 30 °/s and FDI = 500 ms. **(D)** Area under averaged %OIA traces as a function of masking distance for experiments performed with v_T = 10 °/s and FDI = 500 ms. No apparent masking effects in %OIA traces leading to successful collisions at v_T = 10 °/s.

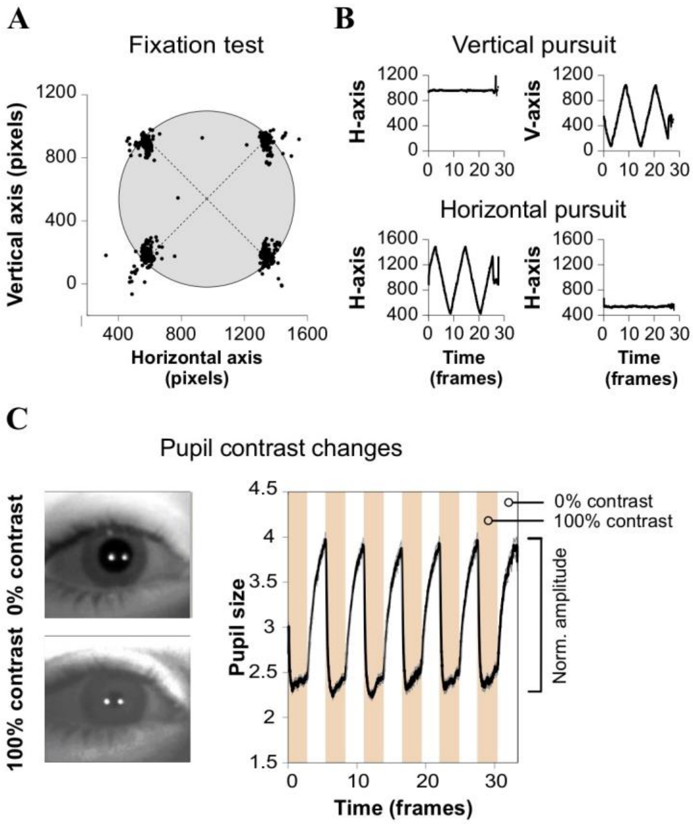

Sup Fig 4. Calibration and standardization of eye-tracking data. (A) Four-point calibration routine illustrated with fixation points positioned on the circular arena's periphery in our visuomotor task (c_2). **(B)** A subsequent calibration involved participants tracking a white dot's horizontal or vertical movement along the circular arena at a speed of 10 °/s (c_3). **(C)** The third calibration assessed pupil size changes when transitioning between a very bright (soft orange background) and a completely black screen (white background). The panel on the right shows average pupillary diameters in response to rapid changes in screen contrast from 0 to 100% (c_4). The response from each participant was recorded separately and served to calibrate further pupillary responses (see **Methods**).

Supplementary References

1. Treviño M, Medina-Coss y León R, Támez S, Beltrán-Navarro B, Verdugo J. Directional uncertainty in chase and escape dynamics. Journal of Experimental Psychology: General. 2024;153: 418–434. doi:10.1037/xge0001510
